# Testing the effects of a prenatal depression preventive intervention on parenting and young children’s self-regulation and functioning (EPIC): protocol for a longitudinal observational study

**DOI:** 10.1186/s12889-021-11385-5

**Published:** 2021-07-10

**Authors:** Alicia Diebold, Jessica K. Johnson, Marianne Brennan, Jody D. Ciolino, Amelie Petitclerc, Lauren S. Wakschlag, Craig F. Garfield, Chen Yeh, Aiko Lovejoy, Dana Zakieh, S. Darius Tandon

**Affiliations:** 1 Institute for Public Health and Medicine, Center for Community Health, Northwestern University Feinberg School of Medicine, 750 N Lake Shore Drive, Suite 680, Chicago, IL 60611 USA; 2 Department of Preventive Medicine-Biostatistics, Northwestern University Feinberg School of Medicine, 680 N Lake Shore Drive, Suite 1400, Chicago, IL 60611 USA; 3 School of Psychology, Laval University, G1V 0A6 Quebec, Canada; 4 Department of Medical Social Sciences, Northwestern University Feinberg School of Medicine, 625 N. Michigan Avenue, Suite 2100, Chicago, IL 60611 USA; 5 Department of Pediatrics, Northwestern University Feinberg School of Medicine, 633 St. Clair Street, Suite 19-059, Chicago, IL 60611 USA; 6 Institute for Innovations in Developmental Sciences, Northwestern University Feinberg School of Medicine, 633 St. Clair Street, Chicago, IL 60611 USA

**Keywords:** Depression, Postpartum, Prevention, Parenting, Child development, Child self-regulation

## Abstract

**Background:**

Perinatal depression is a pervasive public health concern that disproportionately affects low-income women and can have negative impacts on parenting and child developmental outcomes. Few interventions focus on preventing perinatal depression. Previous studies suggest that Mothers and Babies is efficacious in preventing the worsening of depressive symptoms and the onset of postpartum depression. This manuscript presents the protocol of the EPIC study (Effects of a Prenatal Depression Preventive Intervention on parenting and young children’s Self-Regulation and Functioning) to test the effects of Mothers and Babies on parenting and child developmental outcomes through 54 months postpartum. EPIC is an observational study that builds on a completed cluster-randomized trial (CRT). Innovations of this study are direct observations of a subsample of mother-child dyads and the inclusion of fathers/caregivers’ variables as moderators of maternal mental health.

**Methods:**

For this study, we plan to enroll 738 women with children under 30 months old, ≥18 years old, and who speak English or Spanish. Additionally, 429 fathers, partners, or other adult caregivers will be recruited through women participating in the study. Women will be recruited through the parent study (intervention and control participants) or through one of 10 home visiting programs in Illinois (control participants). Data collection will take place through maternal self-report at five time points (when the child is 30, 36, 42, 48, and 54 months), paternal self-report at three time points (when the child is 30, 42, and 54 months), and through mother-child observations at three time points (when the child is 36, 42, and 48 months). Outcome domains include maternal mental health, cognitive-behavioral and parenting skills, and child self-regulation and functioning. Moderators include the contributions of fathers/caregivers, race-ethnicity, and socioeconomic disadvantage. Power and sample size were calculated assuming a two-sided 5% type I error rate and assumed analyses on the individual level.

**Discussion:**

This study has several key strengths and innovations, as well as great potential significance to influence the long-term trajectories of parenting and child development via prenatal intervention.

**Trial registration:**

The study was retrospectively registered at ClinicalTrials.gov (Identifier: NCT04296734) on March 5, 2020.

**Supplementary Information:**

The online version contains supplementary material available at 10.1186/s12889-021-11385-5.

## Background

The prevalence of perinatal depression has been estimated at 10–22% and disproportionately affects women with low-income in all racial and ethnic groups [1–4]. Disparities also exist among women exhibiting clinically relevant depressive symptoms but not meeting DSM criteria for major depression, with studies indicating that rates of depressive symptoms among low-income women are 30–45%—double the rates found among women of higher socioeconomic status [5–8]. Numerous studies have demonstrated that depressed mothers exhibit lower parenting self-efficacy [9, 10], less responsiveness to their child’s cues, and less positive emotion when engaging with their young children [11, 12] compared to non-depressed mothers. Depressed mothers also more frequently engage in hostile or reactive parenting [13]. Negative parenting practices occur among women with elevated but subclinical depressive symptoms, as well as during periods when they are relatively symptom free [14]. The adverse effects of maternal depression on child development have also been well documented, with children of depressed mothers exhibiting greater dysregulation and distress in infancy, and cognitive and language delays as well as emotional, social, and behavioral problems in early childhood [15].

Although parenting interventions have demonstrated some positive effects on parenting behaviors and tend to be associated with a reduction in child behavioral problems among depressed mothers, the limited research with long-term follow-up suggests that effects are rarely sustained over time [16–20]. The limited efficacy of parenting interventions with depressed mothers may be explained by two main reasons. First, typical parenting interventions do not explicitly target mothers’ skills for regulating their thoughts, emotions, and behaviors. Thus, even if they are able to improve their parenting practices in the short term, mothers at risk for depression are likely to revert back to emotional and cognitive patterns that impair their ability to engage in responsive, positive interactions with their child. Given evidence of an association between improvement in maternal depression and improvement in parenting and young child outcomes [21], interventions explicitly focused on promoting maternal mental health may result in greater impact on parenting and child development [22, 23]. Second, parenting interventions often focus on parents of children already exhibiting self-regulatory problems, yet highest impact prevention would occur prior to onset of these difficulties [24, 25]. Recent reviews have hypothesized that prenatal interventions to reduce maternal mood disturbances, in particular, may have beneficial carry-over effects for the child [26, 27]. This research suggests that interventions that begin prenatally and explicitly target maternal mood disturbances should be examined for their effects on both parenting outcomes and child development outcomes.

**Fig. 1:**
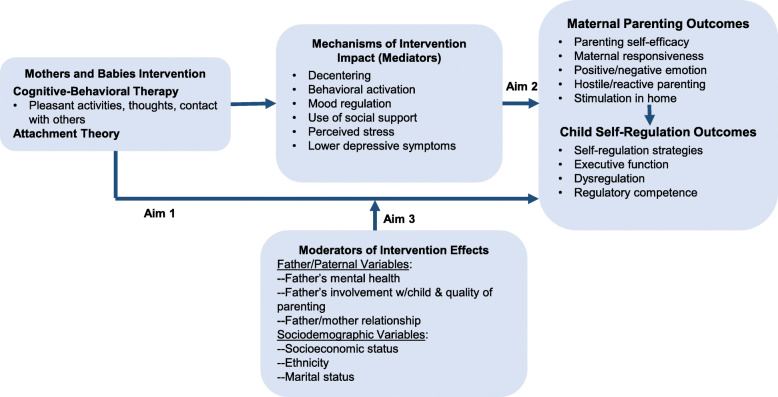
EPIC Conceptual Model

The Effects of a Prenatal Depression Preventive Intervention on parenting and young children’s Self-Regulation and Functioning (EPIC) study examines the effect of an evidence-based perinatal depression prevention program—Mothers and Babies (MB)—on parenting and child development in the preschool years (2.5–4.5 years of age). Building on a completed cluster-randomized trial (CRT) [28, 29] that delivered the MB intervention prenatally to low-income women, the study will examine whether targeting depression symptoms prenatally can prevent the kinds of negative parenting and child development outcomes that so often affect families of depressed mothers. As shown in Fig. 1, a major thrust of MB is fostering development of core cognitive-behavioral therapy (CBT) skills for managing negative emotions and mood, including self-directed and socially oriented regulatory strategies. The specific aims of this study are:

- Aim 1: To test the hypothesis that MB improves parenting and child self-regulatory skill acquisition through 54 months of age. We hypothesize that women who received MB prenatally will exhibit more responsive parenting and their children will show larger increases in self-regulation over the preschool period.
- Aim 2: To test hypotheses about the maternal mechanisms by which MB influences parenting and child self-regulation. We hypothesize that intervention effects will be mediated by core skills targeted by MB, in particular, mothers’ increased awareness of thoughts and feelings, and mothers’ improved mood regulation.
- Aim 3: To test whether fathers’ contributions and key sociodemographic factors moderate MB impact on the mother’s parenting and the child’s self-regulation. We hypothesize that more positive fathers’ mental health, greater father involvement in parenting, and stronger father-mother relationships will enhance MB’s positive effects on mother’s parenting and child outcomes. We will also examine whether maternal minority race/ethnicity, lower socioeconomic status, and adolescent motherhood moderate MB’s preventive effects.

## Methods

### Study design

The EPIC longitudinal study builds on a completed CRT that enrolled 874 pregnant women from home visiting (HV) programs to examine the effects of MB on maternal mental health through 6 months postpartum [29]. The parent study was conducted with 37 HV programs across seven states (Illinois, Minnesota, Ohio, Michigan, Iowa, Missouri, and West Virginia). Of these 37 HV programs, 16 implemented MB using mental health professionals (e.g., licensed clinical social workers), 15 implemented MB using paraprofessional home visitors, and six were control sites delivering usual HV. The parent study’s primary outcome was maternal depressive symptoms as measured by the Quick Inventory of Depressive Symptomatology (QIDS), with secondary outcomes related to core CBT skills—specifically, mood regulation, behavioral activation, modification of harmful thought patterns, and utilization of social support [28, 29]. The EPIC study, a long-term observational cohort study, will re-consent the same cohort of women enrolled in the CRT and recruit women from 10 additional HV programs to serve as additional control participants (receiving usual HV). The children’s fathers/caregivers will also be recruited. The children will be 30 months of age at the first EPIC assessment, and will be followed until 54 months.

### Study participants and recruitment

This study will include mothers enrolled in the parent study, as well as new control mothers, their children, and the children’s fathers/caregivers.

#### Parent study participants

Of the 874 women enrolled in the parent study, after accounting for attrition and lack of interest, we expect to enroll 598 participants whose data may contribute to EPIC—489 who received the MB intervention and 109 who served as controls. Inclusion criteria for the parent study were: 1) > 16 years old, 2) ≤33 weeks’ gestation upon referral, 3) English or Spanish speaking. All women enrolled in the parent study who did not withdraw from the study, decline to be contacted for future studies, or experience a miscarriage or infant loss will be eligible for participation. Participants from the parent study were referred from HV programs that were randomized into one of three study arms: 1) MB delivered by a mental health professional, 2) MB delivered by a paraprofessional home visitor, 3) usual care [28].

#### New control participants

Guided by our power calculations, we will recruit an additional 140 women from 10 Illinois evidence-based HV programs (10 sites at an average of 14 women per site) to serve as additional usual care controls. As a study incentive, HV programs will be offered a free MB training to occur after participant recruitment concludes. Using procedures identical to those used in the parent study, HV program staff will be asked to provide recruitment materials (flyers, brochures) to clients meeting eligibility criteria. Clients will be asked to either contact the research team directly or the HV program will ask the client’s permission to share their contact information with the research team. Women recruited as new controls must be English or Spanish speaking, be > 18 years, and have a child who will turn 30 months old by the end of the recruitment period, which is December 31, 2021. These participants will receive usual care HV services identical to those received by women in the usual care group for the parent study.

#### Fathers/caregivers

A sample of 429 fathers/caregivers will be recruited through existing and new study participants. Since the goal of this data collection is, in part, to obtain additional parent-report data on child development, if the biological father is not available or not actively involved in the upbringing of the child, we will recruit the partner (male or female) or other caregiver (e.g., grandmother) who is currently most involved in the daily life of the child. We will use the following approach to recruit fathers/caregivers. First, we will inform mothers about our interest in recruiting her child’s father (or her partner or another caregiver) into this study, ask the mother’s permission to contact this individual, and obtain current contact information for this person. Second, we will develop recruitment materials that include father-specific language, images, and content supporting their role and importance to the family and the study. Fathers/caregivers recruited into the new cohort must be > 18 years, English or Spanish speaking, and have regular contact (either in-person or virtually) with the child. These participants will not receive any part of the intervention.

All recruitment and consent procedures have been approved by the Northwestern University (NU) Institutional Review Board. Research staff will initially contact a prospective study participant by phone, text, or email and then follow-up with a phone call to explain the research study, ascertain interest, and send a link with an informed consent form (see Additional file 1 for a sample consent form) via a secure web-based application, Research Electronic Data Capture (REDCap) [30] for those agreeing to participate. Research staff will use a standardized script for eligibility screening. For participants who consent to study participation more than 1 month before their child’s 2nd birthday, research staff will contact the participant monthly to remind them about their upcoming 30-month assessment. If research staff are unable to contact a participant by phone, text, or email, an outreach letter will be mailed. For any new referrals (new controls or fathers/caregivers), research staff will follow-up with the referral source (HV program or female study participant respectively) for assistance in contacting the referral. All study materials are available in English and Spanish, and can be administered either orally or in writing based on participant preference.

### Participant outreach and retention strategies

Research staff will obtain as much tracking information as possible during enrollment, including the participant’s name, email address, home and cell phone numbers, Facebook username, mailing address, HV site, and secondary contact information for three people who will always know how to reach a participant. We will update the contact information at each follow-up time point, and confirm each participant’s preferred method of communication. Participants will be given the option to complete surveys over the phone if they are unable or less comfortable completing surveys online. Research staff will stay in regular communication with participants (minimally monthly) throughout the study using the participant’s preferred method of contact and preferred language (English or Spanish). This is similar to the protocol used in the parent study [28].

We will use several strategies to minimize study attrition, including a) using multiple contact modalities (e.g., phone, email, Facebook), b) using contact information for three additional individuals who can assist in reaching the study participant, c) allowing both online and phone survey completion, d) and intensive follow-up from research staff between assessments, including at least monthly phone or text message contact between assessment time points and maternal, paternal, and child birthday cards. Facebook’s direct messaging feature will be used if study team members cannot reach the participant via phone, email, or text. For any participants that no longer have a viable contact method (e.g., phone number out of service), we will run searches using White Pages Premium to obtain updated contact information.

### Intervention conditions

Women in the intervention arms were assigned to receive the six-session MB group intervention delivered by either a mental health professional or home visitor in addition to usual HV services. The six sessions were divided into three two-session modules that correspond with key cognitive-behavioral elements: pleasant activities, thoughts, and social support/contact with others. Integrated throughout MB are activities and skills based on attachment theory that emphasize how each CBT module relates to promoting a strong, nurturing connection between parent and child. Facilitators lead each session using an Instructor’s Manual and each group member had her own Participant Manual. At the end of each group session, a “personal project” was assigned to group members that facilitated skill practice between sessions.

Women in the intervention arms had a varying degree of intervention receipt (0–6 sessions) [29]. While the parent study involved MB delivered in a group setting either via trained mental health professional or a home visiting paraprofessional, we will group all participants receiving intervention into one exposure group, regardless of the deliverer (home visitor vs. mental health professional). Further, since we plan to evaluate whether receipt of MB is associated with the aforementioned outcomes, we plan to define “receipt” as attending at least four of the possible six MB sessions. Primary analyses will thus compare those receiving intervention according to this definition compared to those who did not receive the intervention. Of note, those not receiving the intervention may include participants randomized in the parent study to the MB intervention that did not attend any sessions and/or additionally recruited control participants. The resultant primary independent exposure variable for analyses is binary: attended at least four MB sessions vs. attended zero MB sessions. Fathers/caregivers were not subject to the intervention and were not explicitly engaged during the intervention period.

Women in the parent study’s control condition and newly recruited controls receive usual HV services. All HV programs use an evidence-based model, such as the Parents as Teachers, Healthy Families America, or Early Head Start HV models [31]. These models call for bi-weekly home visits during pregnancy and through a child’s second birthday, with criteria for decreasing or increasing frequency based on achievement of program milestones and client need. During pregnancy, the following areas are addressed during home visits: (a) preparation for childbirth and having a young child in the home, (b) provision of emotional and tangible social support, (c) discussion of infant and young child development, and (d) referrals to community resources for social and health services. During the postpartum period, home visits continue to focus on these same core areas, with an increased focus on encouraging positive parenting and promoting child development and school readiness.

### Data collection

Beginning at 30 months postpartum, we will administer maternal-report surveys every 6 months, continuing until 54 months postpartum with all mothers. In-person observations of parenting and child development will occur in a subset of 115 families at 36, 42, and 48 months (split evenly between intervention and control). We will also conduct father/caregiver-report surveys at 30, 42, and 54 months. Participants will receive a prepaid stored value bankcard as remuneration for each activity.

REDCap [30] will be used for administering surveys and managing study data. Survey links will be sent to participants at scheduled time points, and reminders to complete the surveys will be sent to participants through their preferred method of contact (text, email, phone) until the survey is completed or the deadline for the survey (within 2 months of being sent) is reached, whichever comes first.

The majority of the observational research will occur within participants’ homes in the state of Illinois. For study participants who live in the Chicago-area and prefer not to do observations in their home, they will be given the option of doing the observational component of the study at a developmental laboratory at NU. Observations will be video-recorded by research staff to facilitate coding and assess reliability. Some assessments will be live-scored by a trained assessment specialist and entered into the REDCap database system after the assessment is completed. Coding will be done by trained coders. If a mother does not want to participate in the observational assessments, it will not affect her home visiting or early childhood services, or her ability to participate in the survey component of the study.

### Instruments

Measures were selected based on reliability, validity (for English- and Spanish-speaking populations), and prior use in mental health and HV research with low-income populations. Child outcomes were selected based on developmental sensitivity for early childhood, parallel constructs measurable across survey and direct assessments, and feasibility of the direct assessments within the home-based setting. Mothers will complete surveys assessing maternal mental health, maternal cognitive-behavioral skills, and father/caregiver parenting. Maternal parenting and child developmental outcomes will be assessed by survey and direct observation. Father/caregiver parenting and their perception of child developmental outcomes will be assessed by self-report. Outcomes fall into the following domains: maternal mental health, parenting, child self-regulation, and child functioning. We will explore cognitive-behavioral skills as mediators and paternal mental health, paternal involvement, and contextual influences on child development as moderators. See Table 1 for the full list of instruments and time points for administration.

**Table 1:** SPIRIT Flow Diagram

		STUDY PERIOD					
TIMEPOINT		18–30*	30*	36*	42*	48*	54*
**ENROLMENT:** **Eligibility screen** **Informed consent**							
		X					
		X					
**OUTCOME INDICATOR:**	**ASSESSMENT:**						
**Mental Health and Cognitive-Behavioral Skills**							
Depressive Symptoms	QIDS		XX	X	XX	X	XX
Behavioral Activation	BADS		X	X	X	X	X
Decentering	EQ		X	X	X	X	X
Social Support	MOS-SS		X	X	X	X	X
Perceived Stress	PSS		XX	X	XX	X	XX
Relationship with Partner	RDAS		XX	X	XX	X	XX
**Parenting**							
Parenting Self-Efficacy	PACOTIS		XX	X	XX	X	XX
Maternal Responsiveness	P-COS			**X**	**X**	**X**	
	B-ERA			**X**	**X**	**X**	
Positive and Negative Emotions	P-COS			**X**	**X**	**X**	
	B-ERA			**X**	**X**	**X**	
Hostile/Reactive Parenting	PACOTIS		X	X	X	X	X
	P-COS			**X**	**X**	**X**	
	B-ERA			**X**	**X**	**X**	
Father/Partner Engagement	FRPN Father-Engagement Scale		X*		X*		X*
Paternal Contact	FRPN Father-Child Contact		XX	X	XX	X	XX
**Child Self-Regulation & Functioning**							
Self-Regulation	ECBQ-VS		XX	X	XX	X	XX
	DECA		XX	X	XX	X	XX
Emotion Identification	AKT			**X**	**X**	**X**	
Executive Functioning	MEFS			**X**	**X**	**X**	
Dysregulation	MAP-DB		XX	X	XX	X	XX
	DB-DOS			**X**	**X**	**X**	
School Readiness	Bracken Scale			**X**	**X**	**X**	
Impairment	FLIS		XX	X	XX	X	XX
Contextual Influences on Development	Modified NICHD Measures		X	X	X	X	X

*Months Postpartum, X = Maternal Self-report Only, XX = Maternal and Paternal/Caregiver Self-report, X* = Paternal/Caregiver Self-report Only, **X** = Observation

#### Maternal mental health and cognitive-behavioral skills

Depressive symptoms will be measured by the 16-item Quick Inventory of Depressive Symptomatology (QIDS) [32, 33], which is designed to assess severity of depressive symptoms consistent with DSM-IV symptom criteria. The QIDS scoring system converts responses from the 16 separate items into the nine DSM-IV symptom criterion domains. The total score ranges from 0 to 27. Scores between 0 and 5 = no depression; 6–10 = mild depression; 11–15 = moderate; 16–20 = severe; 21+ = very severe. The QIDS has excellent internal reliability for outpatient samples and its construct validity has been established. Perceived stress will be measured by the Perceived Stress Scale-4 item version (PSS-4), which measures the level to which an individual considers his/her life stressful and how controllable an individual appraises his/her life. Each item contains a 5-point scale ranging from never to very often [34]. Behavioral Activation will be measured using the Behavioral Activation Depression Scale (BADS) [35]. The BADS contains 25 items, each rated on a 7-point scale ranging from 0 (not at all) to 6 (completely), and examines behaviors believed to underlie depression: activation, avoidance/rumination, work/school impairment, and social impairment [35]. Decentering will be measured using the Experiences Questionnaire (EQ) [36]. The EQ is a 20-item self-report scale designed to measure decentering and rumination, which has demonstrated strong internal consistency in a number of studies examining effects of interventions that incorporate cognitive restructuring techniques [37]. Social support will be measured using the 19-item Medical Outcomes Study Social Support Survey (MOS-SSS) [38]. This brief self-administered survey includes an overall functional social support index, as well as four functional support subscales: affectionate, emotional/informational, tangible, and positive social interaction [38]. Relationship with partner will be measured using the 7-item Revised Dyadic Adjustment Scale (RDAS), which measures consensus in decision-making, satisfaction in relations and cohesion. Each item asks respondents to rate certain aspects of their relationship on a 5- or 6-point scale, with higher total scores indicating greater relationship satisfaction [39, 40].

#### Parenting

Mothers and fathers/caregivers will both report on their Parenting self-efficacy using the Parenting Self-Efficacy subscale of the Parental Cognitions and Conduct Toward the Infant Scale (PACOTIS) [41]. They will both report on the amount of Father/Caregiver Contact with the child using the Fatherhood Research & Practice Network (FRPN) Measure of Father-Child Contact, which assesses frequency of play, caregiving, and support from the father figure or other caregiver [42]. They will rate their own Hostile/reactive parenting using the hostile/reactive parenting subscale of the PACOTIS, which measures parents’ and caregivers’ responses to difficult behavior in young children. Fathers/caregivers will rate their level of Engagement with the child using the FRPN Father Engagement scale [43].

Parenting will be assessed via observation in the following ways. Maternal parenting practices will be assessed using the Parenting Clinical Observation Schedule (P-COS) [44] and the Brief Early Relational Assessment (B-ERA). The P-COS is an observational coding system designed to code parent behavior during the parent context of the Disruptive Behavior Diagnostic Observation Schedule (DB-DOS), which is used in this study (see below). The P-COS focuses on three domains of parenting: responsive involvement, problematic discipline, and constructive discipline. The P-COS and its companion child observational schedule, the DB-DOS, have been validated across the preschool period [44–46]. The B-ERA [47] is an observational schedule assessing the affective and behavioral dimensions of the parent-child relationship, including the relational dyad (e.g., mutual enjoyment, reciprocity, tension).

#### Child self-regulation & functioning

Child self-regulation and functioning are assessed by parent report and direct observation of the child. Our assessment battery for child self-regulation is theoretically and empirically driven, and specifically validated for diverse populations in the 30–54 month developmental range, measuring: a) self-regulation, b) executive functioning, c) emotion identification, d) functioning, and e) dysregulation.

Mothers and fathers/caregivers will complete three questionnaires that measure the aforementioned constructs: the Early Childhood Behavior Questionnaire-Very Short Form (ECBQ-VS), Devereaux Early Childhood Assessment (DECA), and Multidimensional Assessment Profile of Disruptive Behavior Short Form (MAP-DB) [48]. The ECBQ-VS is part of the Rothbart suite of temperament questionnaires and generates a self-regulation profile based on child dispositional effortful control, surgency, and negative emotionality [49]. We will use the DECA’s self-regulation and social competence indices, which assess self-regulatory behavior (e.g., accepting other choices) and competent behaviors (e.g., patience). The MAP-DB will assess problem behaviors along a dimensional spectrum. Originally developed to assess transdiagnostic (e.g., irritability) and disruptive behaviors within developmental context, the MAP-DB now also assesses impulsive, depressive and anxious dimensional spectra of behavior [48, 50].

The Family Life Impairment Scale (FLIS) [51, 52] will assess the extent to which children’s emotions and behavior interfere with child functioning, interactions with their family, and functioning in childcare settings.

In addition to parent report, child self-regulation and functioning will be assessed using a battery of direct observation measures, Disruptive Behavior Diagnostic Observation Schedule (DB-DOS), Minnesota Executive Function Scale (MEFS), Affect Knowledge Test-Shortened (AKT-S), and Bracken School Readiness Assessment, Third Edition (BSRA-3). The DB-DOS is a diagnostic observation schedule assessing dysregulation among preschool-aged children via a series of compliance, play and frustration tasks. The full DB-DOS entails three modules: two with an examiner [45] and one with the child’s parent. The current study will use the parent context only, to focus on the child’s self-regulatory capacities in the context of the parental relationship. The MEFS is an adaptive, tablet-based virtual card sort measure of executive function, validated for children 2 years of age and older [53]. The AKT-S is a measure of young children’s emotional knowledge and understanding [54]. The AKT-S has been widely used in research and has demonstrated reliability and validity as a developmentally-appropriate measure of young children’s emotion knowledge. Finally, the BSRA-3 will be used to assess children’s early academic skills. It is a standardized assessment of children’s foundational concept knowledge across five dimensions: colors, numbers, letters, shapes, and sizes/comparisons [55].

#### Contextual influences on child development

We will use a modified version of the National Institute of Child Health and Development’s (NICHD) Study of Early Childcare and Youth Development [56] questions to measure the child’s early childhood education and care experiences (including formal and informal non-maternal childcare, and preschool) and control for their impact on child self-regulation.

### Data management and monitoring

#### Data management

The study surveys will be conducted using web-based surveys, created using REDCap. All study-related information will be kept on password-protected computers only accessible by the research team. Only the research team will have access to individually identifiable private information about human subjects. To safeguard confidentiality and anonymity, all data collection instruments will identify participants only by study ID number. Video-recordings of the observational assessments will be uploaded to the NU research team’s secured NU server; research project shared drive, which is password protected and accessible only by our authorized study personnel within 48 h of observation completion. All data collected during the observational assessments will be entered into REDCap, and scanned and saved onto the shared drive within 48 h of completion. Hard copies will be stored in a locked file cabinet in our locked office location. After the data is entered, a second person will review the data to ensure accuracy of data entry. If a discrepancy is found, a third person will review and resolve the issue.

#### Data monitoring

In the collection of information about depressive symptoms, the research team has the ethical responsibility to appropriately respond to clinical information obtained in this study. The research team will draw on expertise of the investigative team to ensure that staff are well-trained to handle any clinical concerns that arise appropriately and in a sensitive and timely fashion. There are several types of concerns that may arise that will require review and possible referral (1): imminent risk of self-harm and/or potential child abuse and neglect (2); scores indicating clinical concern derived from standardized instruments. Study personnel are required to complete a Mandated Reporter [57] training before conducting any assessments with study participants. If there is reasonable cause to believe a child is being abused or neglected, study team members will be instructed to immediately call the state reporting Hotline and report this to the Principal Investigator. If individuals endorse suicidal ideation, a member of the research team will attempt to reach the study participant by the following business day at the latest and assess the severity of the threat. If the participant reports a threat of imminent self-harm, the research team member will contact 911 and stay on the phone with the participant until they arrive.

Regardless of level of threat, participants will be provided with mental health and parenting resources. These resources will also be provided to participants who score in the ‘severe’ and ‘most severe’ categories on the QIDS assessment. After the research team member ends the call with the study participant, they will call the Project Manager with relevant details, reporting what the client said and endorsed, as well as the information they gathered during the assessment and call with the participant. The research team member will then follow up with an email to the home visitor (if applicable), Project Manager, as well as the Principal Investigator with relevant details. For the observational assessments, a similar protocol will be followed. The Clinical Assessment and Resource Specialist will oversee any clinical concerns that may arise with the children and their families and will follow up with the families if anything of concern comes out of the observational assessments. As this study may be treated as a low-risk, purely observational study, and after assessment of NU’s IRB, we determined the study does not require an external data monitoring committee (DMC).

### Data analysis

Descriptive statistics will summarize baseline characteristics overall and by group (i.e., intervention vs. control). As appropriate, mean ± standard deviation (or median [inner quartile range] will be used in cases of skewed or non-normal empirical distributions) and frequency (proportions) will summarize continuous and categorical data, respectively. We plan to group the MB intervention arms (HV and mental health from the parent study) into a single arm for primary analyses. There will be additional analyses examining the subgroups of participants receiving MB led by HVs (HVP in parent study) versus those led by mental health professionals (MHP arm in parent study), and/or we will explore addition intervention type as a covariate in any modeling.

Our analyses assume normality among our outcomes, but in cases where these assumptions are not borne out, we will transform data and/or use nonparametric analyses. Analyses will occur at the level of the individual participant, with all hypotheses tests assuming a two-sided 5% level of significance unless otherwise specified. We will apply the Benjamini-Hochberg correction [58] to control family-wise type I error rate to 5%. The multiple follow-up time points will allow for longitudinal modeling and trajectory analysis of outcomes over time; however, there are specific study time points of interest (e.g., 30 months and 54 months) for which we estimate specific contrasts across study arms in relevant outcomes along with 95% confidence limits. As the primary analyses of the parent study suggest negligible levels of intracluster (i.e., intra-site) correlation once accounting for participant-level covariates, we do not plan to include a random site effect in analyses. However, we will include random participant (i.e., intercept) effects in all longitudinal models to allow us to distinguish between within and between participant variation, and make for more precise estimation of intervention/fixed effects. We plan to explore multiple methods of analyses to compare across exposure groups (0 sessions vs. 4+ sessions) (1): classic multiple regression methods, whereby we include fixed pre-specified set of covariates in the longitudinal model for outcome(s) (2), inverse probability weighting (IPW) using propensity scores (PS, i.e., the probability of ‘full’ exposure or at least 4 MB sessions) (3), inclusion of PS as a covariate in longitudinal model(s), and (4) pooling study group estimates across PS strata [59–61]. Sensitivity analyses (a) may explore multiple propensity score approaches for multiple levels of intervention (none, partial, complete) [62, 63], and/or (b) add in the participants in the “partial dose” group from the parent study via either random (re) sampling into one of the primary exposure groups (none vs. full dose receipt) or a best/worst case sampling (e.g., add in all those in the partial dose group into either the zero dose or the full dose and repeat analyses).

Sample size and power calculations were completed under the assumption of 20% attrition for primary outcome analyses; thus, while we anticipate missing data, we still expect adequate power to detect differences between study groups under large amounts of missing data. We will look at rates of missing data for all variables and determine whether there are differences in the rates based on participant characteristics, HV program, location, or study arm. These analyses will indicate the extent to which missing data could bias results. We will attempt to collect data at each time point regardless of participants’ level of engagement in earlier data collection efforts. To minimize missing data due to loss to follow-up, we will devote considerable attention to promoting study retention using approaches noted in the research strategy. Mixed effect models to be used in our analyses will account for the possibility of unbalanced data across study time points. However, we plan to apply multiple imputation in order to test for bias introduced by missing data in sensitivity analyses; we will impute at least five datasets to generate an estimated average intervention effect.

Primary parental and child outcomes may be treated as continuous variables. Since assessments occur at five different time points, we will have ability to conduct longitudinal data analysis in order to examine outcome trajectory patterns from 30 to 54 months of age. We first plan to employ a series of mixed models for each relevant outcome (e.g., PACOTIS subscales). Each model will contain fixed intervention (MB intervention received as part of the parent study vs. control) and time effects and random participant effects. In order to address primary hypotheses we will statistically test (against a null hypothesis of no effect) fixed intervention effect coefficients in the mixed model. Regardless of significance, we plan for to adjust all analyses for the following covariates: race/ethnicity, whether a participant is a first-time mother, primary language, education, and mental health service utilization. These variables will be added as fixed effects in the longitudinal models. We may also explore higher-order terms such as interactions, quadratic, etc. Residual diagnostics will allow for assessment of model assumptions and transformations, etc. will be made as appropriate. Any modifications or updates to analyses will be documented in an updated version of the statistical analysis plan (SAP). See Additional file 2 for the current version of the SAP. This version of the SAP focuses on the analyses for Aim 1. Detailed analysis plans for Aims 2 and 3 are forthcoming. See Table 2 for an overview of the analyses of the aims.

**Table 2:** Overview of Study Aim Analyses

Study Aim	Hypothesis	Analysis
1. To test the hypothesis that MB improves parenting and child self-regulatory skill acquisition through 54 months of age.	Women who received MB prenatally will exhibit more responsive parenting and their children will show larger increases in self-regulation over the preschool period.	1. Explore long-term effects of MB on maternal depressive symptom scores. 2. Measure the association between MB receipt and parenting practices. 3. Measure the association between MB receipt and child self-regulation.
2. To test hypotheses about the maternal mechanisms by which MB influences parenting and child self-regulation.	Intervention effects will be mediated by core skills targeted by MB, in particular, mothers’ increased awareness of thoughts and feelings, and mothers’ improved mood regulation.	1. Explore the extent to which mothers’ cognitive-behavioral skills (mediators) affect parenting practices. 2. Explore the extent to which these mediators affect child self-regulation.
3. To test whether fathers’ contributions and key sociodemographic factors moderate MB impact on the mother’s parenting and the child’s self-regulation.	More positive fathers’ mental health, greater father involvement in parenting, and stronger father-mother relationships will enhance MB’s positive effects on mother’s parenting and child outcomes.	1. Examine whether fathers’ mental health, involvement, and relationship with mothers moderate effects on mothers’ parenting practices. 2. Examine whether fathers’ mental health, involvement, and relationship with mothers moderate effects on child self-regulation. 3. Examine whether maternal minority race/ethnicity, lower socioeconomic status, and adolescent motherhood moderate MB’s preventive effects.

We will further explore the use of a semi-parametric, group-based mixture model (SAS PROC TRAJ) [64, 65] to separate distinct longitudinal patterns of outcomes over time (independent of intervention received); we will allow for between two and six trajectory patterns, using Bayesian Information Criteria (BIC) to determine the best fitting model. Upon selection of the final number of trajectory patterns, we will examine whether intervention may predict trajectory membership for the trajectory analyses using generalized linear mixed modeling approaches. We anticipate requiring either multinomial or binomial distributional assumptions with a corresponding logit or generalized logit link in order to predict trajectory membership. We will again explore associations between potential covariates and trajectory membership.

Outcomes measured in the subgroup of participants undergoing more extensive observation at 36, 42 and 48 months of child age (*n* = 115) will be analyzed via the same analytic strategy over these three time points, but the overall sample size and outcomes will be different for this subgroup. Finally, we will perform a separate set of analyses in the group of women for whom we have perinatal and postpartum data up to 6 months postpartum from the parent study. This will allow for a longitudinal model of outcomes collected as part of both studies (e.g., PACOTIS) that includes a wider timespan. We will explore the addition of change-point (i.e., spline) terms as appropriate.

### Exploratory analyses

Regarding the parental responsiveness assessments, we will explore factor analysis using principal component analysis (PCA) for factor extraction on the B-ERA for the observational study sample. These analyses will be deemed exploratory in nature. We further include a set of “lower tiered” study outcomes that are deemed exploratory as well. Depending upon breadth and depth of the primary and secondary analyses, analyses on these additional variables may be included in primary dissemination materials; however, we envision these additional analyses as potentially reserved for downstream dissemination material. Similarly, we may explore subgroup analyses and heterogeneity of effects according to racial/ethnic categories, whether a participants is a first-time mother, primary language, education, and mental health service utilization.

### Power and sample size considerations

Power and sample size calculations assume a two-sided 5% type I error rate and, given the negligible effect of site (or cluster) noted in the parent study, we assume analyses on the individual level. From the parent study data and previous literature, we assume a standard deviation in PACOTIS Self Efficacy (SE) and/or Hostile Reactive Parenting Behavior (HRB) ranging from 1.6 to 1.7 points. Further, while we anticipate the distribution of each of these subscales to be fairly skewed, we view a meaningful difference between two groups to be 0.5 points on the 10-point scale. That is, a change in score from 8.0 to either 8.5 or 7.5 would be meaningful on the SE scale, and a change in score from 1.5 to 2.0 or to 1.0 would be meaningful on the HRB scale.

Table 3 presents power for the primary PACOTIS outcomes under varying sample size scenarios. In each scenario we assume standard deviation = 1.7 units and difference between groups = 0.5 points, on average. Further, we refer to “Intervention” as the full dose exposure group (4–6 sessions) and “Control” as the zero dose exposure group (0 sessions). We assume a 20% dropout rate. Thus, to have between 80 and 90% power, the total target sample size across these two exposure groups should be between 240 and 320 per group of 480–640 total.

**Table 3:** Sample Size and Power Considerations for Primary Analyses: SE and HRB Outcomes

Power	N Intervention (Analysis)	N Intervention (20% LTFU)	N Control (Analysis)	N Control (20% LTFU)	Total N
0.90	256	320	256	320	640
0.85	219	274	219	274	548
0.80	192	240	192	240	480

Sample size estimates for observational assessments were based on the B-ERA measure. Previous data have varying standard deviations that ranged from about 0.5 to 0.8, with both extremes seeming unlikely. We chose to use 0.7 as our guide in sample size and power considerations. Table 4 provides sample size and power calculations for between 0.5–1 point mean difference across exposure groups.

**Table 4:** Sample Size and Power Considerations for Observational Assessments: BERA

Power	Mean Difference	Standard Deviation	N per Group (Analysis)	Total N (Analysis)	Inflate Total for 10% LTFU	Inflate Total for 20% LTFU
0.91	0.5 points	0.7	46	92	102	115
0.86	0.5 points	0.7	39	78	87	98
0.80	0.5 points	0.7	34	68	76	85
0.92	1 point	0.7	13	26	29	33
0.86	1 point	0.7	11	22	24	28
0.81	1 point	0.7	10	20	22	25

## Results

This study is in progress. Recruitment for this study commenced in November 2019, and we anticipate enrollment will continue through December 2021. Through the end of April 2021, we have enrolled 873 participants into this study—485 women from the parent study, 118 new control participants, and 270 fathers/caregivers.

## Discussion

This study has several notable strengths and innovations. First, using multiple propensity-score based methods to complement our classic multiple regression analyses, we will be able to more precisely characterize the relationship between intervention exposure and outcomes, and evaluate the robustness (or lack thereof) of this relationship. Second, the incorporation of fathers and other caregivers provides a more ecologically valid and holistic approach to assess the caregiving environment. Our data collection with fathers/caregivers will allow us to examine the potential moderating role of fathers on MB intervention effects and obtain dual (mother & father/caregiver) perspectives on child self-regulation. The third major strength and innovation of our longitudinal approach (with up to 5 time points from 30 to 54 months) and observational sub-study (performance based assessments for a sub-sample at 36, 42, and 48 months) is its potential for revealing the pattern and sustained nature of MB effects. The large survey sample with multiple time points will enable us to investigate whether children in the intervention group have different growth patterns (e.g., accelerated skill development and reduction in problems) or show sleeper effects, i.e., not evident until more mature self-regulatory capacities are present. The nested observational sub-sample reduces method variance and allows for more precise specification of self-regulation improvements linked to MB. Another novel aspect of our study is the nested multi-method design within a HV program. This enables the power of the full sample with survey measures, while also providing a more nuanced look at these same parenting and child self-regulatory outcomes via direct assessment. Findings will shed light on the added value of these more intensive assessments in detecting intervention effects, important for pragmatic considerations in scaling this approach [66]. Survey and observational assessments include state-of-the art measurement specifically designed for typical:atypical differentiation of normative misbehavior vs. clinical markers in early childhood [50]. Lastly, this study leverages the robust infrastructure of the parent study, which recruited 874 racially and ethnically diverse pregnant women from HV programs.

The observational portion of this study was anticipated to begin in March 2020, however, due to COVID-19 and the subsequent state-issued stay-at-home orders, we were unable to begin this phase of the study. It is currently unclear when it will be safe and feasible to begin in-home observational assessments. The research team, with consultation from the co-investigators and assessment developers, modified the observational assessment protocol to be done entirely remote. The remote protocol was piloted, and at the end of April 2021, 26 remote observational assessments were completed. In response to COVID-19, we also added an additional data collection instrument, a modified version of the Coronavirus Perinatal Impact Survey and Impact Update (COPE-IS/COPE-IU) [67, 68], to collect information on how study participants were impacted by the pandemic.

This study will examine the longer-term effects of a CBT-based group intervention—Mothers and Babies (MB)—on maternal parenting practices and child self-regulation and functioning. This project has great potential significance, as preventive interventions delivered prenatally such as MB may have the ability to influence long-term trajectories of parenting practices and child development which, in turn, can chart a course for future child health and well-being. Prior studies suggest that MB can influence parenting and child outcomes. Although a number of studies have shown post-hoc effects of perinatal intervention on child outcomes [69], studies apriori designed to examine mechanisms of effect on improved child outcomes are far less common [23]. EPIC is designed with power to examine mechanisms by which improved maternal regulation leads to children’s self-regulatory competence, a theorized pathway by which bolstered maternal mental health provides the foundation for a robust adaptive pathway for offspring. The examination of paternal and other contextual moderators will also shed light on the critical but rarely examined role of family ecological factors in this pathway. The larger sample size in this study will provide greater statistical power to detect small to moderate effect sizes, and to examine the potential mediating role of core CBT skills on parenting and child outcomes. Should we find that women receiving MB exhibit more responsive parenting and/or their children exhibit better self-regulation and functioning, there is considerable potential to scale the MB intervention to HV programs across the country, as HV exists in all 50 states. Given the potential implications for research and practice, study findings will be disseminated to researchers interested in perinatal mental health, child/parent interactions, and child self-regulation, as well as HV and early childhood practice communities.

## Supplementary Information

**Additional file 1.** EPIC Sample Consent Form. This consent form is used for participants recruited from the parent study. Similar consent forms are used for participants recruited from new control sites and for fathers/caregivers.

**Additional file 2.** Statistical Analysis Plan. Version 1 of the Statistical Analysis Plan for Effects of a Prenatal Depression Preventive Intervention on Parenting and Young Children’s Self-Regulation and Functioning (EPIC) Study.

## Data Availability

The datasets used and/or analyzed during the current study are available from the corresponding author on reasonable request.

## Abbreviations

CBT: Cognitive-behavioral therapy
CRT: Cluster-randomized trial
HV: Home visiting
IRB: Institutional Review Board
MB: Mothers and Babies
NU: Northwestern University
REDCap: Research Electronic Data Capture
