# Supplementary material for: Testing the effects of a prenatal depression preventive intervention on parenting and young children’s self-regulation and functioning (EPIC): protocol for a longitudinal observational study
Source: BMC Public Health. 2021 Jul 10;21:1368. doi: 10.1186/s12889-021-11385-5 (PMC8271322; doi:10.1186/s12889-021-11385-5)
Supplement: Supplementary file 1 — Additional file 1. EPIC Sample Consent Form. This consent form is used for participants recruited from the parent study. Similar consent forms are used for participants recruited from new control sites and for fathers/caregivers. [file 12889_2021_11385_MOESM1_ESM.pdf]

Online Consent to Participate in Research

Title of Research Study: Effects of a Prenatal Depression Preventive Intervention on Parenting and Young Children's Self-Regulation and Functioning (EPIC)

IRB Study Number: STU00209764

Principal Investigator: S. Darius Tandon, PhD
Center for Community Health, Institute for Public Health and Medicine
Feinberg School of Medicine, Northwestern University, Chicago, IL

Supported By: This research is supported by Northwestern University Institute for Public Health and Medicine and the Eunice Kennedy Shriver National Institute of Child Health and Human Development (NICHD).

Key Information about this research study:

The following is a short summary of this study to help you decide whether to be a part of this study.

The purpose of this study is to see what impact the Mothers and Babies Program has on parenting and early childhood development. You will be asked to complete internet-based surveys at five time points. You may also be asked to participate in in-person observations with your child. We expect that you will be in this research study for approximately two years. The primary risk of participation is feeling emotional or upset when answering certain questions. The main benefit of participation is adding to the knowledge base of how the Mothers and Babies Program can help families.

Why am I being asked to take part in this research study?

You are being asked to take part in this research study because you participated in a previous Mothers and Babies study.

How many people will be in this study?

We expect about 1287 people will be in this research study.

What should I know about a research study?

- Whether or not you take part is up to you.
- You can choose not to take part.
- You can agree to take part and later change your mind.
- Your decision will not be held against you.

If you say that "Yes, you want to be in this research," here is what you will do

- You will be asked to complete internet-based surveys at five time points that ask about your mood, stress, social supports, parenting, and your child's development. If you are unable to complete surveys online, we will conduct the surveys by telephone.
- Surveys will take approximately 60-75 minutes each to complete, and will be conducted when your child is 2.5, 3, 3.5, 4, and 4.5 years old.
- You may also be asked to participate in three observational assessments with your child if you live in the state of Illinois. These could take place in your home (in person or virtually by video-chat) or at a center at Northwestern University if you live in the Chicago-area. These observations will last

approximately 90-120 minutes and will be video-recorded. The video recording is a mandatory part of the observations. Observations with your child will only be done in your presence. During these observations, you will also be asked questions and to engage in activities with your child. You can choose not to take part in the observational assessments and still complete surveys.

- Members of the research team will be in communication with you throughout the research study via phone, e-mail, mail, and/or Facebook.
- The research team may obtain contact information for you from running searches through White Pages Premium,that provides information from public records, and/or the Illinois Department of Healthcare and Family Services OneSource database.

Is there any way being in this study could be bad for me?

You may feel emotional or upset when answering some of the questions.

What happens if I do not want to be in this research or if I say "Yes", but I change my mind later?

Participation in research is voluntary. You can decide to participate or not to participate.

You can decide not to participate in this research or you can start and then decide to leave the research at any time and it will not be held against you. If you withdraw, no more information will be collected from you. We may use the information already collected from you, *unless you submit a written request to the Principal Investigator that your information be withdrawn.*

What happens to the information collected for the research?

Efforts will be made to limit the use and disclosure of your personal information, including research study records, to people who have a need to review this information. We cannot promise complete secrecy. Organizations that may inspect and copy your information include the IRB and other representatives of this institution.

A description of this clinical trial will be available at http://www.ClinicalTrials.gov as required by U.S. Law. This Web site will not include information that can identify you. At most, the Web site will include a summary of the results. You can search this Web site at any time.

> This research is covered by a Certificate of Confidentiality from the National Institutes of Health. The researchers with this Certificate may not disclose or use information or documents that may identify you in any federal, state, or local civil, criminal, administrative, legislative, or other action, suit, or proceeding, or be used as evidence, for example, if there is a court subpoena, unless you have consented for this use. Information or documents protected by this Certificate cannot be disclosed to anyone else who is not connected with the research except, if there is a federal, state, or local law that requires disclosure (such as to report child abuse but not for federal, state, or local civil, criminal, administrative, legislative, or other proceedings, see below); if you have consented to the disclosure or if it is used for other scientific research, as allowed by federal regulations protecting research subjects.
>
> The Certificate cannot be used to refuse a request for information from personnel of the United States federal or state government agency sponsoring the project that is needed for auditing or program evaluation by NICHD which is funding this project. You should understand that a Certificate of Confidentiality does not prevent you from voluntarily releasing information about yourself or your involvement in this research. If you want your research information released to

an insurer, medical care provider, or any other person not connected with the research, you must provide consent to allow the researchers to release it.

The Certificate of Confidentiality will not be used to prevent disclosure as required by federal, state, or local law of child abuse or neglect and/or reports of harm to yourself or others.

This study is being hosted by REDCap and involves a secure connection. Terms of service, addressing confidentiality, may be viewed at https://projectredcap.org/partners/termsofuse/.

Upon receiving results of your surveys, any possible identifiers will be deleted. You will be identified only by a unique subject number. Your contact information will be stored separately from your survey data and is only being collected for communication and payment purposes. All information will be kept on a password protected computer only accessible by the research team. The results of the research study may be published, but your name will not be used.

Data Sharing

De-identified data from this study may be shared with the research community at large to advance science and health. We will remove or code any personal information that could identify you before files are shared with other researchers to ensure that, by current scientific standards and known methods, no one will be able to identify you from the information we share. Despite these measures, we cannot guarantee anonymity of your personal data.

What else do I need to know?

If you agree to take part in this research study, we will provide you with a prepaid stored value gift card for each survey and observation completed. The amount of the gift card will depend on the time point of the survey/observation:

- 2.5 and 3 year survey and 3 year observation: $25 gift card each
- 3.5 year survey and 3.5 year observation: $30 gift card each
- 4 and 4.5 year survey and 4 year observation: $35 gift card each

If you participate in an observational assessment that is conducted virtually by video-chat, you will receive an additional $10 prepaid stored value gift card per time point to support your data usage during the video-chat.

Who can I talk to?

If you have questions, concerns, or complaints, or if you think the research has hurt you, you may contact the Principal Investigator, Darius Tandon, at 312-503-3398 or dtandon@northwestern.edu; or the Research Project Coordinator, Alicia Diebold, at 312-503-4817 or alicia.diebold@northwestern.edu.

This research has been reviewed and approved by an Institutional Review Board ("IRB"). You may talk to them at (312) 503-9338 or irb@northwestern.edu if:

- Your questions, concerns, or complaints are not being answered by the research team.
- You cannot reach the research team.
- You want to talk to someone besides the research team.
- You have questions about your rights as a research participant.
- You want to get information or provide input about this research.

Optional Elements:

Observational Assessments:

If I participate in an observational assessment with my child and information from the study suggests that my child may have some developmental delay (for example, with school readiness or socio-emotional development), I would like Dr. Anderson to discuss this with me and to give me a brief written report if requested. Information from the study is for research purposes only and does not provide a clinical diagnosis.

_____ **I agree** to be contacted by Dr. Anderson

_____ **I do not agree** to be contacted by Dr. Anderson

Consent to be contacted for future studies:

Check one of the following to indicate your choice:

_____ **I agree** to be contacted for another study in the future

_____ **I do not agree** to be contacted for another study in the future

Consent

If you want a copy of this consent for your records, you can print it from the screen.

If you wish to participate, please click the "I Agree" button and you will be taken to the survey.

If you do not wish to participate in this study, please select "I Disagree" or select X in the corner of your browser.
