# Supplementary material for: Testing the effects of a prenatal depression preventive intervention on parenting and young children’s self-regulation and functioning (EPIC): protocol for a longitudinal observational study
Source: BMC Public Health. 2021 Jul 10;21:1368. doi: 10.1186/s12889-021-11385-5 (PMC8271322; doi:10.1186/s12889-021-11385-5)
Supplement: Supplementary file 2 — Additional file 2. Statistical Analysis Plan. Version 1 of the Statistical Analysis Plan for Effects of a Prenatal Depression Preventive Intervention on Parenting and Young Children’s Self-Regulation and Functioning (EPIC) Study. [file 12889_2021_11385_MOESM2_ESM.pdf]

STATISTICAL ANALYSIS PLAN (SAP)

Effects of a Prenatal Depression Preventive Intervention on Parenting and Young Children's Self-Regulation and Functioning (EPIC)

May 6, 2021

Version 2.0

STATISTICAL ANALYSIS PLAN (SAP)

Principal Investigator: Darius Tandon, PhD
Co-Investigator / Biostatistician: Jody D. Ciolino, PhD

1. INTRODUCTION

This document outlines the proposed primary analyses for the EPIC research protocol. The study will build upon a parent cluster-randomized study funded by the Patient-Centered Outcomes Research Institute (PCORI), examining the efficacy of the group-based Mothers and Babies (MB) intervention in comparison to a control condition. The parent study has three arms (control, MB lead by mental health professionals [MHP], and MB led by home visiting paraprofessional [HVP]) with unequal (1:3:3) allocation. The parent study will follow women and their children from the perinatal period through six months postpartum, and the current study will extend follow-up to 24-48 months of age, with scheduled assessments at 30, 36, 42, 48, and 54 months of age for children born to the women of the MB parent study (in addition to control women recruited during the course of the study). The purpose of this document is to provide detail regarding the statistical analysis plan (SAP) for this study.

As of August 2020, the parent study includes 37 HV sites in the Midwest Region of the United States contributing study data: six control sites, and 16 sites administering intervention delivered via mental health professionals (MHP) and 15 delivering intervention via home visiting paraprofessionals (HVP) (i.e., 31 total sites delivering active intervention). The parent study enrolled (randomized) 874 study participants, 824 of which were included in analyses. We estimate a fraction of these original participants will contribute to the proposed long-term follow-up study. Since the current study has a disproportionate number of control sites, EPIC extension will recruit additional control participants in order to achieve adequate power to address the study aims.

<u>Aims</u>

For the purposes of this SAP, we will focus on aims outlined below for analyses. They stem from the proposed “Aim 1” of the EPIC Research Strategy: **To examine whether MB improves parenting practices and child self-regulation through four-five years of age**.

Specifically, these analyses aim:

1. To explore **long-term effects** (through 4.5 years) of MB on **maternal depressive symptom scores** as measured by the 16-item Quick Inventory of Depressive Symptomology (QIDS). We hypothesize that overall depression symptom scores will, on average, be lower for women who received the MB intervention.
2. To **measure the association** between **MB receipt and parenting practices**.
    a. We hypothesize that those women who received MB intervention will, on average, exhibit improved parenting practices in comparison to those women who did not receive MB.
    b. Parenting behaviors will be measured via both **self-report** (whole sample) and **observational assessments** (subset sample).
3. To **measure the association** between **MB receipt and child self-regulation**.
    a. We hypothesize that children of mothers receiving the MB intervention will, on average, exhibit greater signs of self-regulation compared to those of mothers not receiving the intervention.
    b. Child behavior will be measured via both **parent report** (whole sample) and **observational assessments** (subset sample).

Assessment time points for the EPIC study include: Months 30, 36, 42, 48, and 54 for all self-reported assessments. Observational assessment time points for a subset of the study sample include: Months 36, 42, and 48.

2. OUTCOMES

Outcome are listed below for each objective. For each objective, we reserve a 0.05 type I error in primary analyses:

1. **Depressive symptoms** are measured by the 16-item Quick Inventory of Depressive Symptomatology (**QIDS**), total score ranging from 0 to 27 points. Of note, scores on QIDS map to differing depression severity levels as follows:
   a. 0-5: no depression
   b. 6-10: mild depression
   c. 11-15: moderate depression
   d. 16-20: severe depression
   e. 21-27: very severe depression

   This outcome will be treated as continuous for primary analyses; however, secondary and exploratory analyses may involve (a) categorization of this outcome according to severity categories above, and/or (b) restricting analyses to a subsect of participants according to severity categories above. Primary analyses will be conducted at the two-sided 5% level of significance. Secondary analyses will involve a correction for multiple hypotheses as appropriate.

2. **Parenting outcomes** of interest include:
   a. Primary:
      i. Parenting **self-efficacy (SE)** will be measured via the Parental Cognitions and Conduct Toward the Infant Scale **(PACOTIS)**: mean of items 5, 7, 9, 11, 14; scores range from 0-10. Of note, we use an abbreviated scale in our study and SE will be measured via items 1, 11, 2, 4, 7, and 10 in our REDCap-specific surveys.
      ii. Parenting **hostile reactive behavior (HRB)** will also be measured via the PACOTIS: mean of items 8, 10, 12, 15, 18, 22. In our REDCap-specific surveys, HRB will be measured via items 8, 5, 9, 12, 6, and 3.

   Since each of these outcomes above carry equal weight and they test slightly different hypotheses, we plan for a two-sided 5% level of significance for each corresponding analysis. Secondary outcome analyses for this objective (below) will involve correction for multiple hypotheses as appropriate.

   b. **Secondary**: We will focus on the Brief Early Relationship Assessment **(BERA)** observational assessment to measure parenting behaviors. The BERA is a 38-item measure, and it has a multiple factor structure.
      i. Previous analyses suggest the first principal component, or first factor, driving the majority of the variability in this outcome measures **Parental Positive Affect, Sensitive Responsiveness, and Verbalizations**. Items loading heavily on this factor were:

1) Positive, Enthusiastic Affect and Behavior	12) Structures and Mediates Environment
2) Lack of Constricted, Withdrawn, Depressed Affect and Behavior	13) Parent Reads Child's Cues or Behavior and Responds Sensitively
6) Positive, Warm, Encouraging Attitude Toward Child	14) Connectedness
9) Amount and Quality of Visual Contact with Child	15) Mirroring
10) Quality and Amount of Verbalizations	18) Consistency, Predictability

   ii. We will use the **sum score on these 10 items to measure parental responsiveness overall**. This will serve as the major outcome of interest among this subset of sample.
   iii. Additionally, we will conduct a factor analysis using **principal component analysis (PCA)** for factor extraction on the BERA for the observational study sample.
      1. Given the sum score above is broader than we would expect if we were measuring maternal responsiveness, we plan to conduct factor analyses to determine whether a factor emerges that is primarily driven by maternal responsiveness.
      2. If a difference factor structure from those referenced above emerges, we will consider developing a secondary summary score for parenting responsiveness.

3. **<u>Child self-regulation outcomes</u>**
   a. **Primary** outcomes to assess child self-regulation on the entire study sample will be determined by:
      i. The **Early Childhood Behavior** Questionnaire-Very Short Form (**ECBQ-VS**) **Effortful control**: sum of items 21, 27, 31, 8, 15, 35, 7, 14R, 12R, 29, 5, 28
      ii. **Multidimensional Assessment Profile** of Disruptive Behavior Short Form (**MAP-DB**) **Temper Loss Subscale** (sum of 22 temper loss items)

   Since each of these outcomes above carry equal weight and they test slightly different hypotheses, we plan for a two-sided 5% level of significance for each corresponding analysis. Secondary outcome analyses for this objective (below) will involve correction for multiple hypotheses as appropriate.
   b. **Secondary** outcomes include
      i. **Devereaux Early Childhood Assessment (DECA)** according to parent report with the following subscales:
         1. **Initiative**
         2. **Attachment/relationships**
         3. **Self-Regulations**
      ii. The **Disruptive Diagnostic Observation Schedule (DB-DOS)** will be completed for the **subset of observational assessments**. For these analyses we will focus on **Problems in Behavioral Regulation.**
   c. Exploratory outcomes include
      i. **Overall Disruptive Behavior** score from the **MAP-DB**
      ii. ECBQ-VS Negative affect: sum of items 16, 17, 19, 32, 2, 26, 10, 22, 23, 1, 33, 34R (reverse scored)
      iii. ECBQ-VS Surgency: sum of items 4, 13, 18, 20, 24, 6, 11, 9, 25, 3, 30, 36
4. **<u>Additional outcomes reserved for separate SAP(s)</u>**
   There are several additional outcomes we capture throughout the course of the study. The details of the hypotheses and corresponding analyses will be reserved for a separate analysis plan, and the findings related to these outcomes will be reported in separate dissemination materials (i.e., not necessarily included in the primary results dissemination manuscripts). For reference, we list them here:

**Assessment**	**Month 30**	**Month 36***	**Month 42**	**Month 48***	**Month 54**
**Self-Reported Assessments**					
Behavioral Activation (BADS)^	x	x	x	x	x
Decentering (Experiences)^	x	x	x	x	x
Social Support (MOS-SSS)^	x	x	x	x	x
Perceived Stress (PSS-4)	x	x	x	x	x
Dyadic Adjustment (RDAS)	x	x	x	x	x
FLIS	x	x	x	x	x
Father Engagement – fathers only	x		x		x
Father/Child Contact	x	x	x	x	x
**Observational Assessments**					
AKT		x	x	x	
Bracken		x	x	x	

P-COS		x	x	x	
MEFS		x	x	x	

*Mothers only, fathers/caregivers not assessed at these time points
^Mothers only, fathers/caregivers do not complete these assessments

3. DEMOGRAPHICS AND BASELINE ASSESSMENTS

The variables listed below are suspected to be related to outcome assessments. Thus, the study team *a priori* decided to adjust analyses for these potential confounders:

1. Race/ethnicity (at the participant level): minority vs. not belonging to a minority category.
2. Number of children: continuous/count variable for number of children at baseline.
3. Primary language of intervention receipt (for those receiving intervention) or primary language in which the participant completed assessments (for control participants).
4. Education: at least some college vs. less than college.
5. Mental health service utilization: an indicator for enrollment in counseling with a therapist base OR medication for depression at baseline (or both).

Additional demographics that will be summarized in general include:

6. Age.
7. Marital status.
8. Employment status.

4. PRIMARY EXPOSURE VARIABLE

While the parent study involved Mothers and Babies (MB) delivered via a group setting either via trained mental health professional or a home visiting paraprofessional, **we will group all participants receiving intervention into one exposure group, regardless of the deliverer** (home visitor vs. mental health professional). Further, since we plan to evaluate whether receipt of MB is associated with the aforementioned outcomes, we plan to define **"receipt" as attending at least four of the possible six MB sessions.** Primary analyses will thus compare those receiving intervention according to this definition compared to those who did not receive the intervention. Of note, **those not receiving intervention may include participants randomized in the parent study to the MB intervention that did not attend any sessions and/or additionally recruited control participants**. The resultant primary independent exposure variable for analyses is binary: attended at least four MB sessions vs. attended zero MB sessions.

Those participants who received a "partial dose" (i.e., attending between one and three MB sessions) will initially be excluded from the primary analyses, but we will include them in sensitivity analyses to evaluate robustness or sensitivity of results. At the time of this SAP creation, there were 79 enrolled participants who fall into the "partial" dose category (between 1-3 sessions), 217 in the "full dose" category (between 4-6 sessions), and nearly 260 participants in the control group (0 sessions [n=75] or newly recruited [n=185]).

5. DATA STORAGE

Data will be collected and managed using Research Electronic Data Capture (REDCap) housed at Northwestern University's Clinical and Translational Sciences Institute (CTSA), NUCATS (1). REDCap is a secure, web-based application designed for research studies that provides an intuitive interface for validated data entry, audit trails for tracking data manipulation and export procedures, and automated export procedures for seamless data downloads to common statistical packages, and procedures for importing data from external sources.

6. STATISTICAL METHODS

Descriptive statistics will summarize baseline characteristics overall and by group (i.e., intervention vs. control). As appropriate, mean ± standard deviation (or median [inner quartile range] will be used in cases of skewed or

non-normal empirical distributions) and frequency (proportions) will summarize continuous and categorical data, respectively. We plan to group the MB intervention arms (HV and mental health from the parent study) into a single arm for primary analyses. There will be additional analyses examining the subgroups of participants receiving MB led by HVs (HVP in parent study) versus those led by mental health professionals (MHP arm in parent study), and/or we will explore addition intervention type as a covariate in any modeling.

Analyses will employ normal theory methodology as appropriate, and in cases of violations of assumptions, transformations and/or nonparametric analyses may be utilized. Analyses will proceed at the participant level, and all hypothesis tests will assume a two-sided 5% level of significance as above unless otherwise specified. We will apply the Benjamini-Hochberg correction to control family-wise type I error rate to 5% (2). The multiple follow-up time points will allow for longitudinal modeling and trajectory analysis of outcomes over time; however, there are specific study time points of interest (e.g., 30 months and 54 months) for which we estimate specific contrasts across study arms in relevant outcomes along with 95% confidence limits. As the primary analyses of the parent study suggest negligible levels of intracluster (i.e., intra-site) correlation once accounting for participant-level covariates, we do not plan to include a random site effect in analyses. However, we will include random participant (i.e., intercept) effects in all longitudinal models to allow us to distinguish between within and between participant variation, and make for more precise estimation of intervention/fixed effects. We plan to explore multiple methods of analyses to compare across study groups: (1) classic multiple regression methods, whereby we include fixed covariates in the longitudinal model for outcome(s), (2) inverse probability weighting (IPW) using propensity scores (PS), (3) inclusion of PS as a covariate in longitudinal model(s), and (4) pooling study group estimates across PS strata (3) (4, 5). Sensitivity analyses (a) may explore multiple propensity score approaches for multiple levels of intervention (none, partial, complete)(6, 7), and/or (b) add in the participants in the "partial dose" group from the parent study via either random (re)sampling into one of the primary exposure groups (none vs. full dose receipt) or a best/worst case sampling (e.g., add in all those in the partial dose group into either the zero dose or the full dose and repeat analyses).

Sample size and power calculations were completed under the assumption of 20% attrition for primary outcome analyses; thus, while we anticipate missing data, we still expect adequate power to detect differences between study groups under large amounts of missing data. We will examine rates of missing data for all variables and determine whether the rates vary by participant characteristics, HV program, location, or study arm. These analyses will indicate the extent to which missing data could bias results. We will attempt to collect data at each time point regardless of participants' level of engagement in earlier data collection efforts. To minimize missing data due to loss to follow-up, we will devote considerable attention to promoting study retention using approaches noted in in the research strategy. The mixed effects models planned for analysis are generally robust for unbalanced data across study time points. However, we plan to apply multiple imputation in order to test for bias introduced by missing data in sensitivity analyses; we will impute at least five datasets to generate an estimated average intervention effect.

As mentioned in the outcomes and general considerations sections, parental and child outcomes may be treated as continuous variables. Since assessments occur at five different time points, we will have ability to conduct longitudinal data analysis in order to examine outcome trajectory patterns from 30 to 54 months of age. We first plan to employ a series of mixed models for each relevant outcome (e.g., PACOTIS subscales). Each model will contain fixed intervention (MB intervention received as part of the parent study vs. control) and time effects and random participant effects. In order to address primary hypotheses we will statistically test (against a null hypothesis of no effect) fixed intervention effect coefficients in the mixed model. Regardless of significance, we plan for adjusted analyses based on the five covariates listed above. These variables will be added as fixed effects in the longitudinal models. We may also explore higher-order terms such as interactions, quadratic, etc. Residual diagnostics will allow for assessment of model assumptions and transformations, etc. will be made as appropriate. Any modifications or updates to analyses will be documented in an updated version of this SAP.

We will further explore the use of a semi-parametric, group-based mixture model (SAS PROC TRAJ)(8) (9) to separate distinct longitudinal patterns of outcomes over time (independent of intervention received); we will

allow for between two and six trajectory patterns, using Bayesian Information Criteria (BIC) to determine the best fitting model. Upon selection of the final number of trajectory patterns, we will examine whether intervention may predict trajectory membership for the trajectory analyses using generalized linear mixed modeling approaches. We anticipate requiring either multinomial or binomial distributional assumptions with a corresponding logit or generalized logit link in order to predict trajectory membership. We will again explore associations between potential covariates and trajectory membership.

Outcomes relevant to the subgroup of participants undergoing more extensive observation (at fewer study time points, however) will be analyzed via the same analytic strategy, but the overall sample size and outcomes will be different for this subgroup. Finally, we will perform a separate set of analyses in the group of women for whom we have perinatal and postpartum data up to six months from the parent study. This will allow for a longitudinal model of outcomes collected as part of both studies (e.g., PACOTIS) that includes a wider timespan. We will explore the addition of change-point (i.e., spline) terms as appropriate.

Exploratory Analyses
As mentioned above regarding the parental responsiveness assessments, we will explore factor analysis using principal component analysis (PCA) for factor extraction on the BERA for the observational study sample. These analyses will be deemed exploratory in nature. We further include a set of "lower tiered" study outcomes that are deemed exploratory as well. Depending upon breadth and depth of the primary and secondary analyses, analyses on these additional variables may be included in primary dissemination materials; however, we envision these additional analyses as potentially reserved for downstream dissemination material. Similarly, we may explore subgroup analyses and heterogeneity of effects according to racial/ethnic categories, whether a participant is a first-time mother, primary language, education, and mental health service utilization.

7. POWER AND SAMPLE SIZE CONSIDERATIONS

Power and sample size calculations assume a two-sided 5% type I error rate, and given the negligible effect of site (or cluster) noted in the parent study, we assume analyses on the individual level. From the parent study data and previous literature, we assume a standard deviation in PACOTIS Self Efficacy (SE) and/or Hostile Reactive Parenting Behavior (HRB) ranging from 1.6 to 1.7 points. Further, while we anticipate the distribution of each of these subscales to be fairly skewed, we view a meaningful difference between two groups to be 0.5 points on the 10-point scale. That is, a change in score from 8.0 to either 8.5 or 7.5 would be meaningful on the SE scale, and a change in score from 1.5 to 2.0 or to 1.0 would be meaningful on the HRB scale.

Table 1 presents power for the primary PACOTIS outcomes under varying sample size scenarios. In each scenario we assume standard deviation = 1.7 units and difference between groups = 0.5 points, on average. Further, we refer to "Intervention" as the full dose exposure group (4-6 sessions) and "Control" as the zero dose exposure group (0 sessions). We assume a 20% dropout rate. Thus, to have between 80%-90% power, the total target sample size across these two exposure groups should be between 240-320 per group of 480-640 total.

Table 1. Sample Size and Power Considerations for Primary Analyses: SE and HRB Outcomes

Power	N Intervention (Analysis)	N Intervention (20% LTFU)	N Control (Analysis)	N Control (20% LTFU)	Total N
0.90	256	320	256	320	640
0.85	219	274	219	274	548
0.80	192	240	192	240	480

Sample size estimates for observational assessments were based on the BERA measure. Previous data have varying standard deviations that ranged from about 0.5 to 0.8, with both extremes seeming unlikely. We chose to use 0.7 as our guide in sample size and power considerations. To Table 2 provides sample size and power calculations for between 0.5-1 point mean difference across exposure groups.

Table 2. Sample Size and Power Considerations for Observational Assessments: BERA

Power	Mean Difference	Standard Deviation	N per Group (Analysis)	Total N (Analysis)	Inflate Total for 10% LTFU	Inflate Total for 20% LTFU
0.91	0.5 points	0.7	46	92	102	115
0.86	0.5 points	0.7	39	78	87	98
0.80	0.5 points	0.7	34	68	76	85
0.92	1 point	0.7	13	26	29	33
0.86	1 point	0.7	11	22	24	28
0.81	1 point	0.7	10	20	22	25

8. TECHNICAL DETAILS

The SAP is subject to version control, and we anticipate modifications to analytic plans be documented herein. As in any study, the analytic plan may change due to assumption violations, logistical issues, unexpected empirical distributions of study outcomes, or a combination thereof. In these cases, the SAP will be updated accordingly. All analyses will be performed via SAS version 9.4 or higher (The SAS Institute; Cary, NC) or R version 4.0.4 or higher (The R Foundation for Statistical Computing platform). Table and figure formatting and style may be dictated by mode of dissemination or specific target journal(s) for results dissemination.

While there are no formal interim analyses planned, the statistical team will develop data status and quality reports to monitor data quality, completeness, and parameter assumptions throughout the course of the study. There will be no interim analyses involving statistical hypothesis testing, and any unplanned analyses will be documented as such in any dissemination materials.

References:

1. Harris PA, Taylor R, Thielke R, Payne J, Gonzalez N, Conde JG. Research electronic data capture (REDCap)—a metadata-driven methodology and workflow process for providing translational research informatics support. Journal of biomedical informatics. 2009;42(2):377-81.
2. Benjamini Y, Hochberg Y. Controlling the false discovery rate: a practical and powerful approach to multiple testing. Journal of the Royal statistical society: series B (Methodological). 1995;57(1):289-300.
3. Austin PC. An introduction to propensity score methods for reducing the effects of confounding in observational studies. Multivariate behavioral research. 2011;46(3):399-424.
4. Leyrat C, Caille A, Donner A, Giraudeau B. Propensity scores used for analysis of cluster randomized trials with selection bias: a simulation study. Statistics in medicine. 2013;32(19):3357-72.
5. Leyrat C, Caille A, Donner A, Giraudeau B. Propensity score methods for estimating relative risks in cluster randomized trials with low-incidence binary outcomes and selection bias. Statistics in medicine. 2014;33(20):3556-75.
6. Spreeuwenberg MD, Bartak A, Croon MA, Hagenaars JA, Busschbach JJ, Andrea H, et al. The multiple propensity score as control for bias in the comparison of more than two treatment arms: an introduction from a case study in mental health. Medical care. 2010:166-74.
7. McCaffrey DF, Griffin BA, Almirall D, Slaughter ME, Ramchand R, Burgette LF. A tutorial on propensity score estimation for multiple treatments using generalized boosted models. Statistics in medicine. 2013;32(19):3388-414.
8. Jones BL, Nagin DS. Advances in group-based trajectory modeling and an SAS procedure for estimating them. Sociological methods & research. 2007;35(4):542-71.
9. Jones BL, Nagin DS, Roeder K. A SAS procedure based on mixture models for estimating developmental trajectories. Sociological methods & research. 2001;29(3):374-93.
